# Foam Rolling vs. Proprioceptive Neuromuscular Facilitation Stretching in the Hamstring Flexibility of Amateur Athletes: Control Trials

**DOI:** 10.3390/ijerph20021439

**Published:** 2023-01-12

**Authors:** Albert Pérez-Bellmunt, Oriol Casasayas-Cos, Paolo Ragazzi, Jacobo Rodríguez-Sanz, César Hidalgo-García, Max Canet-Vintró, Iván Caballero-Martínez, Laura Pacheco, Carlos López-de-Celis

**Affiliations:** 1 Faculty of Medicine and Health Sciences, Universitat Internacional de Catalunya, 08017 Sant Cugat del Vallès, Spain; 2 ACTIUM Functional Anatomy Group, Universitat Internacional de Catalunya, 08195 Sant Cugat del Vallès, Spain; 3 Departamento de Fisiatría y Enfermería, Unidad de Investigación en Fisioterapia, Facultad de Ciencias de la Salud, Universidad de Zaragoza, C/Domingo Miral, s/n, 50009 Zaragoza, Spain; 4 Institut Universitari per a la Recerca a I’Atenció Primària de Salut Jordi Gol i Gurina (IDIAPJGol), 08007 Barcelona, Spain

**Keywords:** extensibility, healthy adults, ROM, muscle stretching, hamstring muscles, strength, flexibility, fascia, myofascial rolling

## Abstract

Background: the use of stretching techniques in the sports world is frequent and common thanks to their many effects. One of the main benefits of stretching is an increased range of motion (ROM). Recently, the use of a foam roller has spread in sports practice due to benefits that are similar to those of shoes observed in stretching. The objective of the following study was to compare the results of proprioceptive neuromuscular facilitation stretching (PNF) with foam rolling (FR). Methods: The design of the study was a single-blind, randomized controlled trial (clinicaltrial.gov NCT05134883), and the participants were 80 healthy young athletes. The range of motion was evaluated with a modified sit-and-reach test before, during (at 30 s), and at the end of the intervention (at 2 min). The subject’s discomfort sensation was measured using the Borg scale. Effect sizes were calculated using Cohen’s d coefficient. Volunteers were randomized into the PNF group or FR group. Results: the differences were statistically significant (*p* < 0.001) during the intervention in favor of PNF group. The differences at the end of intervention showed that the PNF group had a greater increase in flexibility, with this difference being statically significant (*p* < 0.001). The sensation of perceived exertion with PNF at the end of the intervention was similarly classified as moderate for both groups. Conclusion: Despite the fact that the use of FR is spreading in the field of sports and rehabilitation, the results of the present study suggest that the gain in flexibility in the hamstrings is greater if PNF-type stretches are used instead of FR.

## 1. Introduction

Hamstring muscle injuries are frequent in many sports and are associated with a high rate of recurrence [1,2], especially when fast acceleration or high-speed running is required [3,4]. Moreover, in certain sports such as soccer, the recidivism rate for hamstring injuries is 12–31% [2,5]. Therefore, the etiology of hamstring injuries has been extensively researched and documented.

Although there is no consensus in the literature [6], some studies suggest that a combination of abnormalities (strength, flexibility, warm up, fatigue) or morphological proximal attachment increase the risk of hamstring strain [7,8]. Moreover, poor hamstring flexibility has been associated with pelvic, knee, and lower back pain [9,10], and it may affect sport performance [11]. For these reasons, proper hamstring stretching is the key to flexibility improvement in these muscles, with it being part of many rehabilitation and training programs in the health or sport fields [12].

Traditionally, stretching techniques have been used to improve flexibility, with an understanding of this property as the range of motion (ROM) availed in a joint. Although there is no widespread consensus on what type of stretching is the most effective, the literature appears to report a greater increase in the ROM in the case of proprioceptive neuromuscular facilitation (PNF) stretching techniques when compared to other stretching techniques [13,14,15,16].

Another popular technique in rehabilitation and fitness to increase the ROM is foam rolling (FR), also known in the literature as self-roller release or myofascial rolling. This flexibility effect of FR can be local or distant [17]. FR is a collective term for manual therapy techniques based on the application of mechanical force using a foam roller on soft body tissue. Recent studies have showed that a single FR application can increase the ROM of a joint immediately after the treatment [18,19,20] and for durations of up to 30 min post-treatment [21]. Although an increased ROM is the main effect of FR [22,23], muscular recovery after exercise [24] and sport performance [25,26] have also been investigated.

Previous research comparing the acute effects of stretching and foam rolling on the ROM have either reported no difference between stretching and FR [27], a favorable effect regarding FR versus stretching techniques [28], or a favorable effect regarding stretching versus FR on the ROM [29]. A recent systematic review [18] showed that the effects of stretching techniques and FR on the ROM were very similar. However, this paper is focused on the effects of foam rolling and not in the comparison between these two techniques. A more recent meta-analysis focused on comparing these two techniques [20]. This current research reports similar effects on the ROM concerning stretching and FR, but this paper does not divide the results by stretching type. Furthermore, the time application of the PNF and stretching was not equal and was very different from that used in the clinical field. Due to all of these reasons, the aim of this study was to compare the acute effect on the ROM between FR and two different PNF interventions with different durations.

## 2. Materials and Methods

### 2.1. Study Design

The study was a single-blind randomized control trial (https://clinicaltrials.gov, accessed on 30 January 2022, NCT05134883). Interventions and measurements were carried out by the same three blinded therapists, with one controlling the intervention and the other taking measurements and recording data. To minimize bias, participants could not see and were not informed of any result obtained during measurement. A local committee approved this study (CBAS). Data collections was carried out according to the international ethical standards for humans of the Declaration of Helsinki [30].

### 2.2. Participants

**Figure 1:**
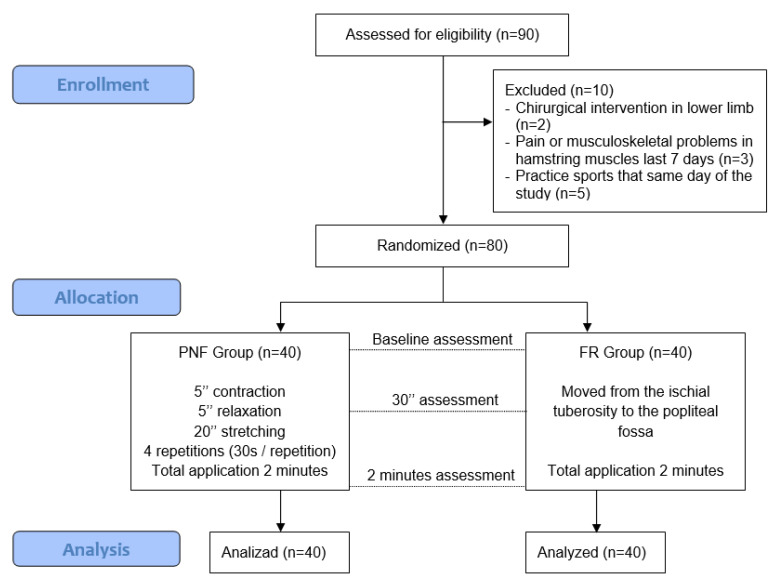
Enrollment. Stages of the study. PNF, proprioceptive neuromuscular facilitation stretching; FR, foam rolling.

Participants were recruited from the university community. Ninety healthy volunteers were initially screened and ten were excluded using different exclusion criteria (Figure 1). Finally, the final sample was composed of 80 subjects. A flow chart of patient recruitment and retention is presented in Figure 1, which displays the process from initial contact to the analysis of the results. The written informed consent of the subjects was obtained prior to baseline examination.

A pilot study was conducted in order to calculate the sample size. The variable used for the sample size calculation was the improvement in hamstring muscle flexibility. In a previous pilot study carried out in our population, the standard deviation around the mean hamstring muscle flexibility was 10.3 mm and the difference was 6.5 mm at baseline. Using this data, 40 patients per extremity were needed for a 5% confidence level and 80% statistical power.

The inclusion criteria stated that the subject needed to be over 18 years old. The exclusion criteria consisted of hypermobility, hamstring injury in the previous 6 months, diagnosed orthopedic problems or surgery in the lower limbs, back pain or spine surgery, and systemic or neurological disorders.

Interventions and measurements were carried out by the same three blinded therapists, two of which controlled the intervention, with the other taking measurements and recording data. To minimize bias, participants could not see and were not informed of any result obtained during measurement.

The same conditions, such as the temperature, equipment, and time of day, were used in all the cases.

After baseline examination, patients were randomly assigned to one of two groups: PNF or FR. The allocation sequence was determined before the study using a computer-generated randomization list (Ramdom.org).

### 2.3. Variables and Measurements

ROM of hip. To evaluate the hip ROM, hamstring flexibility (mm) was evaluated with a modified sit-and-reach test (MSR) before, during (at 30 s), and at the end of the intervention (at 2 min). This test was selected based on its wide use by clinicians to evaluate hamstring flexibility and its good correlation coefficient [31,32,33]. It was carried out following the recommendations of previously published research [32,34]. The volunteers sat on the floor with their lower limbs stretched out and together; their backs and hips were supported against the wall (90° hip flexion), and the soles of their feet were placed against the edge of a box. Participants then extended their arms forward, placing the same hand on top of the other with both hands facing down and with their back against the wall at all times. They then reached forward, sliding their hands along the measuring scale as far as possible without bending their knees [35].

**Figure 2:**
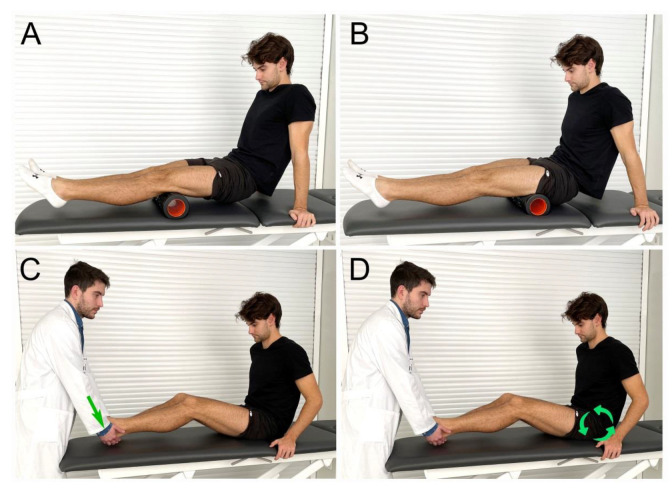
Intervention protocol and data collection. (**A**,**B**), foam rolling intervention; (**C**,**D**), proprioceptive neuromuscular facilitation stretching intervention; green arrow, force versus resistance exerted by the patient; green arrows in circle, pelvic anteversion movement performed by patient.

The subject’s discomfort sensation was measured using the Borg rating of perceived exertion scale (RPE). The participants were asked at the end of the intervention to give a number from 1 to 10, were 1 was the easiest and 10 was the hardest (Figure 2).

### 2.4. Interventions

After enrollment, the participants signed an informed consent form, and each participant was randomly assigned to one of the following two groups.

The PNF group. A PNF stretching protocol was performed with each participant assuming a long sitting position on a plinth with their knees maintained as extended as possible. At this moment, the participant was asked to perform maximum isometric hamstring muscle contraction for 5 s followed by 5 s of relaxation and 20 s of stretching (Figure 2). The therapist showed the participant how to maintain this isometric contraction with the flexion of the hip and ankle. Each participant underwent four repetitions of PNF stretching (30 s/rep). The protocol and timings used were taken from previously published work by Esnault and Viel [36]. The hamstring length was recorded at baseline, at the end of the first repetition, and at the end of the protocol (Figure 2).

The FR group. For the application of foam rolling, the subjects assumed a long sitting position on a firm and even surface by placing the arms backward and transferring their body weight to their palms. The foam roller, applied bilaterally, was placed under the hamstrings and slowly moved back and forth from the ischial tuberosity to the popliteal fossa by applying pressure for 2 min (Figure 2). The intervention followed the methodology of previous investigations [37], and the applied pressure was the maximum tolerated by the patients (pushing till discomfort) with no pain [38]. The foam roller was constructed using a hollow PVC pipe (500 DURO) surrounded by neoprene foam (1-cm thickness). The flexibility was measured using the same technique that the PNF group used, i.e., at baseline, during the intervention (30 s from the start of intervention), and at the end of the intervention (2 min). When measurements were taken after 30 s, the time was stopped until the measurement was finished. This way the intervention time was always the same for both interventions.

### 2.5. Statistical Analysis

The statistical analysis was performed using IBM SPSS v.20.0. Descriptive statistics were calculated for all variables. Frequencies were calculated for qualitative variables. Quantitative variables and their differences were expressed as mean and standard de-viation (SD). Prior to the use of the parametric test, the normality assumption was eval-uated using the Shapiro–Wilk test. Levene’s test was used to test for homogeneity of variances between groups. The two-way ANOVA was used to compare between-group and within-group changes over the three measurement periods. This model was performed for each dependent variable where the FR group or PNF group was the between-subjects factor and time was the within-subjects factor. If the assumption of sphericity was violated, the Greenhouse–Geisser correction was utilized for interpretation. When a statistically significant effect was noted, a post-hoc analysis was performed, and the Bonferroni correction was used to adjust for multiple comparisons. Effect sizes (ES) were calculated using Cohen’s d coefficient [39]. The effect size was considered large when ES > 0.8, with around 0.5 being moderate and <0.2 small.

## 3. Results

The sample consisted of 53 men (66.25%) and 27 women (33.75%), with an average age of 22.82 years (4.33 SD). Table 1 shows the descriptive characteristics of the initial sample and group homogeneity.

There were no significant differences between the experimental and control groups at baseline (*p* > 0.05 for all measuring tests). There were significant main effects for time (F = 226.360 (*p* < 0.001)) but not for group (F = 0.302 (*p* < 0.586)). There was significant interaction between group and time (F = 14.545 (*p* < 0.001)). Intra-group analysis showed an increase in hamstring flexibility measured in millimeters, statistically significant in both groups both during the intervention (*p* < 0.001) and 2 min after the intervention (*p* < 0.001). In the foam roller group, we can observe that 47% of the total gain was obtained during the intervention, while 52% of the gain was obtained in the remaining 90 s. In the PNF group, we can observe that 56% of the total gain was obtained during the intervention (30 s), while the remaining 43.5% was obtained by applying three more repetitions of the stretch.

The effect size of the PNF group was higher than that of the FR group both during the intervention and at the end of the intervention (Table 2). When comparing the two groups, it was observed that the PNF group obtained better results that are statistically significant, both during the intervention and at the end of the intervention (Table 3).

When analyzing the sensation of perceived exertion, it was observed that the sensation was similar in both groups (FR group: 4.05 ± 2.48 and PNF group: 4.4 ± 2.21). There was no statistically significant difference between the increased effort and the flexibility gained (*p* = 0.359).

## 4. Discussion

This investigation compared the effect of two popular techniques used to improve the range of motion. The present results show a statistically significant increase in flexibility in the PNF and FR groups and in both periods of intervention (30 s and 2 min both during and at the end of the intervention). When comparing the two groups, this study obtained better and significant results for the PNF group. These results disagree slightly with two recent meta-analyses that had compared the impact of a single bout of stretching with foam rolling on the range of motion [18,26]. Although in both cases the interventions were equally effective, the results of these meta-analyses had shown no significant differences between both groups. These results could be different from ours because these meta-analyses had included all kinds of stretching techniques. The literature presents a greater increase in the range of motion (ROM) as a result of PNF stretching techniques when compared to other stretching techniques [13,14,15,16].

Other studies observed an increase (practically double) in the gain of flexibility in hamstring muscles when following a six sessions program of static stretching compared to FR [40]. A previous study compared the effects of FR to the effects of static stretching on passive ankle dorsiflexion ROM with similar results [41]. On the other hand, Su et al. observed that the use of a foam roller is more effective than stretching to increase the flexibility of the quadriceps and hamstrings [28], but in this case the stretching was a combination of static and dynamic protocols. More recently, research revealed no significant differences between FR and dynamic stretching [42]. In this case, the methodology of stretching and zone of application was different to the present study. When the studies used vibration, the results increased considerably [18].

After 2 min of intervention, the present results show a moderate effect in the case of the PNF group and a smaller effect in the case of FR. For this reason, the results suggest that the application of FR for less than two minutes may not be enough in the field of sports to improve the ROM.

Although this is purely speculative, both protocols likely result in therapeutic effects via different physiological mechanisms. On one hand, a recent paper suggested that the efficacy of PNF in terms of a gain of flexibility is due to the fact that this type of stretching is focused on the musculotendinous unit and its autogenic inhibition reflex [43]. This reflex is produced when a stretching force is applied, with the Golgi organ registering an increase in tension in the muscle tendon and provoking a reflex relaxation of the muscle [15]. On the other hand, the potential mechanism of action of self-myofascial release is focused on the fascia [44]. Certain theories have proposed that the increment of temperature when a foam roller is used facilitates the liberation and deformation of fascial adhesions between muscle layers [22]. This could also be due to the activation of the diffuse noxious inhibitory control (DNIC) mechanism [45]. In this mechanism, the mechanical and painful stimuli of the foam roller is perceived as non-harmful and produces the liberation of pain-relieving endorphins and a reduction in muscle stiffness.

This study has some important limitations. Firstly, this research only analyzed one parameter of neuromuscular response or function (in this case, the ROM). For future studies, it could be interesting to observe viscoelastic or contractile muscle properties. Secondly, the intervention of FR did not use the vibration information; the use of this mechanical oscillation could produce different effects in the muscle tissue. Thirdly, the present study only analyzed the acute effect of these techniques. Fourthly, the results of the present research could have been different of diverse time domains or different muscle applications had been employed.

Finally, our results may also help to provide new insight into athletes’ healthcare and performances by showing how the application of PNF or FR is useful for improving the ROM and how the manipulation of time may promote greater results.

## 5. Conclusions

Despite the fact that the use of FR to obtain more flexibility for hamstrings muscles is growing in the field of sports and rehabilitation, the results of the present study suggest that the gain in the flexibility of the hamstrings is greater if PNF-type stretches are used instead of FR. Despite the results, the use of FR may be beneficial for other processes such as a reduction in muscle tension, blood flow activation and improvements in muscle recovery.

## Figures and Tables

**Table 1:** Shows the descriptive characteristics of the initial sample and group homogeneity. Initial descriptive statistics.

	PNF Group	FR Group
Sex		
Men	29 (72.5%)	24 (60%)
Women	11 (27.5%)	16 (40%)
Age	23.38 (4.24)	22.5 (4.54)
Flexibility	22.48 (10.36)	22.3 (11.29)

Abbreviations: PNF, proprioceptive neuromuscular facilitation Stretching; FR, foam rolling.

**Table 2:** Hamstring flexibility. Intra-group analysis.

	SR-0	SR-1					SR-2				
			Difference SR-0 to SR-1					Difference SR-0 to SR-2			
Variables	Mean ± SD	Mean ± SD	Mean	95% CI	*p*	ES	Mean ± SD	Mean	95% CI	*p*	ES
FR group	22.35 ± 11.30	24.02 ± 11.31	1.67	[0.940; 2.410]	<0.001	0.15	25.90 ± 10.87	3.55	[2.700; 4.400]	<0.001	0.32
PNF group	22.48 ± 10.36	25.67 ± 9.92	3.19	[2.395; 30.990]	<0.001	0.32	28.12 ± 9.71	5.64	[4.772; 6.509]	<0.001	0.56

Abbreviations: SD, standard deviation; ES, effect size; CI, confidence interval; SR-0, baseline assessment; SR-1, during intervention; SR-2, end of intervention; PNF, proprioceptive neuromuscular facilitation stretching; FR, foam rolling.

**Table 3:** Hamstring flexibility. Inter-group analysis.

	Difference between SR-0 and SR-1	Difference between SR-0 and SR-2	Difference between SR-1 and SR-2
	Mean ± SD	Mean ± SD	Mean ± SD
FR group	1.67 ± 1.85	3.55 ± 2.14	1.87 ± 1.30
PNF group	3.19 ± 2.01	5.64 ± 2.19	2.44 ± 1.47
*p*-value	0.001	0.070	0.001

Abbreviations: SR, modified sit-and-reach test; SR-0, Baseline assessment; SR-1, During intervention; SR-2, End of intervention; PNF, Proprioceptive Neuromuscular Facilitation Stretching; FR, foam rolling.

## Data Availability

The data presented in this study are available on request from the corresponding author.
